# A Novel, Single Algorithm Approach to Predict Acenocoumarol Dose Based on *CYP2C9* and *VKORC1* Allele Variants

**DOI:** 10.1371/journal.pone.0011210

**Published:** 2010-06-18

**Authors:** Zoraida Verde, Jonatan R. Ruiz, Catalina Santiago, Beatriz Valle, Fernando Bandrés, Elpidio Calvo, Alejandro Lucía, Félix Gómez Gallego

**Affiliations:** 1 Universidad Europea de Madrid, Madrid, Spain; 2 Unit for Preventive Nutrition, Department of Biosciences and Nutrition at NOVUM, Karolinska Institutet, Huddinge, Sweden; 3 Hospital Clínico de San Carlos, Madrid, Spain; 4 Fundación Tejerina, Madrid, Spain; University Hospital Vall d'Hebron, Spain

## Abstract

The identification of *CYP2C9* and *VKORC1* genes has strongly stimulated the research on pharmacogenetics of coumarins in the last decade. We assessed the combined influence of *CYP2C9* *2 and *3, and *VKORC1* c.-1639G>A, 497C>G, and 1173C>T variants, on acenocoumarol dosage using a novel algorithm approach, in 193 outpatients who had achieved stable anticoagulation. We constructed an “acenocoumarol-dose genotype score” (AGS, maximum score = 100) based on the number of alleles associated with higher acenocoumarol dosage carried by each subject for each polymorphism. The mean AGS was higher in the high-dose (>28mg/week) compared with the low-dose (<7mg/week) group (mean(SEM) of 84.1±3.4 *vs.* 62.2±4.8, P = 0.008). An AGS>70 was associated with an increased odds ratio (OR) of requiring high acenocoumarol dosage (OR: 3.347; 95%CI: 1.112–10.075; P = 0.032). In summary, although more research is necessary in other patient cohorts, and this algorithm should be replicated in an independent sample, our data suggest that the AGS algorithm could be used to help discriminating patients requiring high acenocoumarol doses to achieve stable anti-coagulation.

## Introduction

Oral anticoagulant therapy with vitamin K antagonists (coumarins) is used worldwide for the treatment and prevention of thrombotic diseases. The clinical use of oral anticoagulant therapy is complicated by narrow therapeutic range and the large inter-individual variation that exists in response to the same dosage.

While an insufficient dose may fail to prevent thromboembolism, an overdose increases the risk of serious adverse effects such as intra-cerebral bleeding. Although several factors including gender, age or race have been traditionally considered to explain individual variability in coumarin dosage needed to achieve stable and effective anticoagulation, growing evidence supports the influence of genetic factors [1]. The identification of the genes encoding cytochrome P-450 2C9 enzyme (*CYP2C9*), the principal metabolizing enzyme of the coumarins, and vitamin K epoxide reductase enzyme (*VKORC1*), the molecular target for coumarins, has strongly stimulated the research on pharmacogenetics of coumarins in the last decade. Several variants in CYP2C9 (CYP2C9*2 and especially the CYP2C9*3 allele) and *VKORC1* genes (especially the 1639G>A polymorphism) are associated with effective coumarin derivative dose [2], [3], [4], [5], [6], [7], [8], [9], [10], [11]. *CYP2C9 and VKORC1* seem the only genes with relevant effects on coumarin response [10], except the rs2108622 polymorphism in the gene encoding cytochrome P450, family 4, subfamily F, polypeptide 2 (*CYP4F2*) which could also influence warfarin dose [9]. This enables to set the definitive demarcation of the genetic information that is needed for developing useful coumarin dosing algorithms. With regards to this, a simple recent algorithm to account for the putative influence of several gene variants, and of the complex interaction between these variants, on a given phenotype is the so-called ‘total genotype score’ (TGS). For instance, Kathiresan *et al* [12] recently computed a TGS of nine validated genotypes associated with modulation in blood lipid levels. They showed that the polygenic profile they obtained with the TGS was an independent risk factor for incident cardiovascular disease [12].

The purpose of our study was to assess the *combined* influence of several variants alleles of the *CYP2C9* (*2 and *3) and *VKORC1* (c.-1639G>A, 497C>G, and 1173C>T) polymorphisms, using the TGS approach, on the acenocoumarol dosage needed to achieve stable, effective anticoagulation. We chose the aforementioned gene variants based on their high frequency among Caucasians [13] and on previous research showing their association with acenocoumarol dose [2]. Briefly, the risk of requiring a low dose of acenocoumarol is significantly higher in patients carrying at least one CYP2C9*3 allele, and the *KVORC1* genotypes c.-1639 AA, 497 CG and GG, and 1173 TT [2].

## Results

The genotype frequencies of *CYP2C9* and *VKORC1* genes for the three study groups (low-, medium- and high-dose) are shown in Table 1. The distribution of individuals in the low-, medium- and high-dose groups with genotypes of very slow, slow and normal metabolism for 0 up to 5 polymorphisms is shown in Table 2.

**Table 1:** Genotypes of *CYP2C9* and *VKORC1* genes, genotype score (GS) for each polymorphism, and acenocoumarol dose.

Gene	Polymorphism	GS	Low-dose (%)	Medium-dose (%)	High-dose (%)
*CYP2C9* (Cytochrome P-450 2C9)	CYP2C9*2 or 430C>T (rs1799853)	0 = TT, 1 = CT, 2 = CC	0, 32.4, 67.6	2.8, 33.3, 63.8	5.6, 11.1, 83.3
	CYP2C9*3 or 1075A>C (rs1057910)	0 = CC, 1 = AC, 2 = AA	14.7, 29.4, 55.9	1.4, 15.6, 83.0	0, 0, 100
*VKORC1* (Vitamin K epoxide reductase)	c.-1639G>A (rs9923231)	0 = AA, 1 = GA, 2 = GG	38.2, 35.3, 26.5	21.3, 44.7, 34.0	5.6, 44.4, 35.0
	497T>G (rs2884737)	0 = GG, 1 = TG, 2 = TT	15.2, 39.4, 45.5	10.0, 39.3, 50.7	5.9, 29.4, 64.7
	1173C>T (rs9934438)	0 = TT, 1 = CT, 2 = CC	23.5, 35.3, 41.2	14.2, 46.8, 39.0	5.6, 44.4, 50.0

The group comparisons of genotypic and allelic frequency of these polymorphisms have been published elsewhere [2].

Values of GS: 0 = very slow metaboliser (low dose), 1 = slow metaboliser (medium dose), and 2 = normal metaboliser (high dose).

**Table 2:** Distribution of individuals in the low-, medium- and high-dose groups with genotypes of very low, low and normal metabolism for 0 up to 5 polymorphisms.

Number of genotypes	Low-dose (n = 34)	Medium-dose (n = 142)	High-dose (n = 18)	P value
Very low				
0	50.0 (17)	73.8 (104)	83.3 (15)	0.005
1	20.6 (7)	10.6 (15)	11.1 (2)	
2	17.6 (6)	8.5 (12)	5.6 (1)	
3	11.8 (4)	6.4 (9)	0	
4	0	0.7 (1)	0	
5	0	0	0	
Low				
0	22.0 (8)	22.0 (31)	33.3 (6)	0.242
1	23.5 (8)	26.2 (37)	22.2 (4)	
2	23.5 (8)	17.7 (25)	27.8 (5)	
3	17.6 (6)	19.9 (28)	16.7 (3)	
4	11.8 (4)	12.8 (18)	0	
5	0	1.4 (2)	0	
Normal				
0	2.9 (1)	1.4 (2)	0 (0)	0.006
1	32.4 (11)	21.3 (30)	5.6 (1)	
2	20.6 (7)	29.8 (42)	22.2 (4)	
3	20.6 (7)	14.9 (21)	22.2 (4)	
4	17.6 (6)	18.4 (26)	22.2 (4)	
5	5.9 (2)	14.2 (20)	27.8 (5)	

Values are % (n).

**Figure 1:**
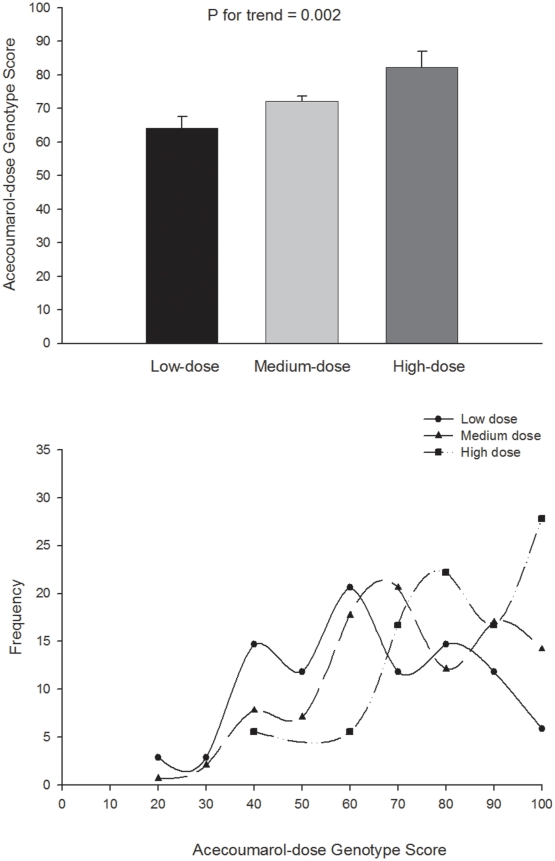
Acenoumarol-dose Genotype Score and drug dose. Upper panel: Acenoumarol-dose Genotype Score (0–100) by dose categories. Values are means and standard error. Post-hoc comparisons indicated that the AGS was significantly lower (P = 0.008) in low-dose vs. high-dose group. Lower panel: Frequency distribution of Acecoumarol-dose Genotype Score in the low-, medium-, and high-dose groups.

The mean AGS was significantly higher (P = 0.008) in the high-dose group compared with the low-dose group (Figure 1). A total of five (29.4%) individuals in the high-dose group had an AGS of 100, versus 2 (6.1%) and 20 (14.3%) in the low- and medium-dose groups respectively. A total of 21 patients per group (high-dose and low-dose group, n = 42 in total) would have been needed in order to demonstrate statistically significant between-group differences in AGS, with a power of 95% and α of 0.05.

The ROC analysis showed a significant discriminating accuracy of AGS in identifying an individual with low-dose (AUC = 0.630; 95%CI: 0.557–0.699, P = 0.009; sensitivity = 0.668, specificity = 0.545) and high-dose (AUC = 0.676; 95%CI: 0.605–0.742, P = 0.017; sensitivity = 0.583, specificity = 0.706). The corresponding AGS cut-off value for low-dose was ≤60, and for high-dose >70. Logistic regression analysis showed that subjects with AGS ≤60 had an increased odds ratio (OR) of being in a need of low-dose of acecoumarol (OR: 2.356; 95%CI: 1.094–5.073; P = 0.028), whereas subjects with AGS >70 had an increased odds ratio (OR) of being in a need of high-dose of acecoumarol (OR: 3.347; 95%CI: 1.112–10.075; P = 0.032). The results did not change after controlling for drugs that can interfere with the anticoagulant effect of acenocoumarol (data not shown).

## Discussion

This is the first report assessing the *cumulative* effects of *CYP2C9* and *VKORC1* variants on the coumarine dose needed to achieve stable anticoagulation using a novel simple algorithm approach, which we labeled ‘AGS’. This method was especially useful to predict outlier patients needing high doses (OR = 3.3347 for those with AGS>70).

The question addressed here is of clinical relevance. Coumarin anticoagulants are among the most widely prescribed drugs. A difficulty with their use is that dosage needs to be individually determined for each patient, usually by following a standard initial dosing protocol and measuring the coagulation rate (e.g., the INR) regularly. Although the current oral coumarin anticoagulants (warfarin, acenocoumarol and phenprocoumon) are likely to be replaced by other drugs, such as specific thrombin inhibitors, the coumarins will probably continue to be the main oral anticoagulants prescribed in the short-medium term. Thus, understanding the factors that can explain individual variability in the coumarin dosage needed to achieve stable anticoagulation is of medical interest. Genetics is one of such factors. In fact, the US Food and Drug Administration recently included reference to *CYP2C9* and *VKORC1* in the prescription information on warfarin [17].

The combined influence of *CYP2C9* and *VKORC1* genotypes accounts for as much as ∼30% of the variability in coumarin dose requirement [1]. Thus, there is currently intense debate as to whether pharmacogenetic algorithms for estimating the dose of coumarins provide a more accurate dose than the fixed-dose approach that is commonly used worldwide [1]. It has also been suggested that the greatest benefit of pharmacogenetic algorithms is observed in patients with extreme dose requirements [8]. With regards to this, the AGS value was significantly higher in our high-dose (>28mg/wk) compared with the low dose group (<7mg/wk), with the ROC analysis showing an AGS cut-off value for low-dose of ≤60, and for high-dose >70. It is also noteworthy that the mean acenocoumarol dosage was 26mg/wk in those outpatients (N = 27) with an AGS of 100, *vs*. 14.5mg/wk in the rest of participants.

Our study is not without potential limitations. Besides the small sample size of our cohort, our findings are limited, at least partly, by the fact that it was not possible to replicate our data in a different cohort of patients, particularly using a prospective design with patients recruited at the start of their treatment. To increase the applicability of genetic algorithms as the one proposed here, further research should study large population cohorts, and include different ethnic groups.

In summary, while recognizing the need for more research in this area, we propose that, in those settings where genomics tool are routinely applied, the AGS algorithm could be used to help discriminating patients requiring high acenocoumarol doses to achieve stable anti-coagulation (*vs.* those requiring low doses), and thus to prevent the serious risks associated with insufficient drug use.

## Methods

The study comprised 193 outpatients [100 men and 93 women; mean (SEM) age: 64 (1) yr]. They all had achieved a stable anticoagulation state at the moment of entering the study (after receiving oral anticoagulant therapy with acenocoumarol within the previous 6+ months), i.e. target INR of 3.0–4.0 in patients with a prosthetic heart valve and of 2.0–3.0 for the rest of patients. They were free of any concomitant severe disease known to interfere with acenocoumarol treatment [2].

We extracted genomic DNA from peripheral EDTA-treated anti-coagulated blood to genotype: (i) *CYP2C9* *2 and *3, and *VKORC1* 3730G>A using real-time polymerase chain reaction (PCR) followed by melting curve analysis with fluorescence resonance energy transfer probes; and (ii) *VKORC1* polymorphisms c.-1639G>A, 497C>G, and 1173C>T with specific PCR followed by single base extension using capillary electrophoresis [2]. The study was approved by our institutional committee (*Universidad Europea de Madrid*, Spain) and all participants provided their written informed consent.

We computed the combined influence of all the five studied polymorphisms following the TGS method described elsewhere [14], [15]. First, we scored each genotype within each polymorphism (Table 1). We assumed an additive model (equaling 0, 1 or 2), that is, on the basis of the number of alleles associated with higher acenocoumarol dosage that were carried by each subject for each polymorphism (Table 1). Thus, we assigned a genotype score (GS) of 2, 1 and 0 to each individual genotype associated with highest, medium and lowest acenocoumarol dose respectively. For instance, a GS of ‘2’ was given for the wild type (CC) genotype of the *CYP2C9* *2 allele (i.e. ‘normal metaboliser’, associated with highest drug dose) *vs.* a GS of ‘1’ for the CT genotype (‘slow metaboliser’, medium dose), and a GS of ‘0’ for the TT genotype (‘very slow metaboliser’, lowest dose).

Second, we summed the GS of each single genotype (GS*_CYP2C9_*_ *2_ + GS*_CYP2C9_*_ *3_ + GS*_VKORC1_*_ c.-1639G>A_ + GS *_VKORC1_*_ 497C>G_ + GS *_VKORC1_*_ 1173C>T_), which allowed us to construct an ‘acenocoumarol-dose genotype score’ (AGS).

Third, the obtained AGS was ‘normalised’ (i.e. transformed to the scale of 0–100) for easier interpretation, as follows:

AGS = (100/10)×(GS*_CYP2C9_*_ *2_ + GS*_CYP2C9_*_ *3_ + GS*_VKORC1_*_ c.-1639G>A_ + GS *_VKORC1_*_ 497C>G_ + GS *_VKORC1_*_ 1173C>T_) [14], [15], where 10 is the result of multiplying 5 (number of studied polymorphisms) by 2, which is the score given to the genotype associated with the highest drug dose. An AGS of 100 represents the theoretically highest possible score for the polygenic profile of acenocoumarol dose, that is, that all GS are 2. In contrast, an AGS of 0 represents the theoretically ‘less acenocoumarol-dose dependent’ profile.

### Statistical analysis

We compared the AGS means among groups of low-, medium- and high-acenocoumarol dose with one-way analysis of covariance, where AGS was entered as a dependent variable, group (low-, medium-, and high-dose) as fixed factor, and sex and age as covariates. We used Bonferroni *post hoc* test for between-group comparisons. We repeated the analysis after further controlling for drugs that can interfere with the anticoagulant effect of acenocoumarol, i.e. ACE inhibitors, angiotensin II receptor antagonists, statins, beta-blockers, Ca^2+^ antagonists, anti aggregants, digoxin, valproic acid, anticonvulsant drugs that are enzymatic inductors (phenytoin, carbamazepine and phenobarbital) and amiodarone.

We compared the distribution of individuals in the low, medium- and high-dose groups with genotypes of very slow, slow and normal metabolism for 0 up to 5 polymorphisms by using the χ^2^ test. The ability of the AGS to correctly classify individuals in the high-dose group (coded as 1) from those in the low- or medium-dose (coded as 0) was analyzed by receiver operating characteristic (ROC) curves [16]. Similarly, we repeated the same procedure to study the diagnostic value of the AGS on identifying individuals in the low-dose (coded a 1) group from those in the medium- or high-dose (coded as 0). We calculated the area under the ROC curve (AUC) and 95% confidence intervals (95%CI). Finally, we used binary logistic regression to study the relationship between AGS and low or high dose.
